# Comparative Effectiveness of Cognitive Behavioral Therapies in Schizophrenia and Schizoaffective Disorder: A Systematic Review and Meta-Regression Analysis

**DOI:** 10.3390/jcm14155521

**Published:** 2025-08-05

**Authors:** Vasilios Karageorgiou, Ioannis Michopoulos, Evdoxia Tsigkaropoulou

**Affiliations:** 1 Second Department of Psychiatry, University of Athens, 12462 Athens, Greece; imihopou@med.uoa.gr; 2 First Department of Psychiatry, University of Athens, 11528 Athens, Greece; evitsigaropoulou@yahoo.com

**Keywords:** cognitive behavioral therapy, psychosis, schizoaffective disorder, affective psychosis, meta-analysis, efficacy, meta-regression, psychotherapy

## Abstract

**Background:** Cognitive behavioral therapy (CBT) has shown consistent efficacy in individuals with psychosis, as supported by many trials. One classical distinction is that between affective and non-affective psychosis. Few studies have specifically examined the possible moderating role of substantial affective elements. In this systematic review and meta-regression analysis, we assess how CBT response differs across the affective spectrum in psychosis. **Methods:** We included studies assessing various CBT modalities, including third-wave therapies, administered in people with psychosis. The study protocol is published in the Open Science Framework. Meta-regression was conducted to assess whether the proportion of participants with affective psychosis (AP), as proxied by a documented diagnosis of schizoaffective (SZA) disorder, moderated CBT efficacy across positive, negative, and depressive symptom domains. **Results:** The literature search identified 4457 records, of which 39 studies were included. The median proportion of SZA disorder participants was 17%, with a total of 422 AP participants represented. Meta-regression showed a trend toward lower CBT efficacy for positive symptoms with a higher SZA disorder proportion (β = +0.10 SMD per 10% increase in AP; *p* = 0.12), though it was not statistically significant. No significant associations were found for negative (β = +0.05; *p* = 0.73) or depressive symptoms (β = −0.02; *p* = 0.78). Heterogeneity was substantial across all models (I^2^ ranging from 54% to 80%), and funnel plot asymmetry was observed in negative and depressive symptoms, indicating possible publication bias. Risk of bias assessment showed the anticipated inherent difficulty of psychotherapies in blinding and possibly dropout rates affecting some studies. **Conclusions:** Affective symptoms may reduce the effectiveness of CBT for positive symptoms in psychotic disorders, although the findings did not reach statistical significance. Other patient-level characteristics in psychosis could indicate which patients can benefit most from CBT modalities.

## 1. Introduction

The spectrum of psychotic disorders encompasses a broad range of diagnoses, most notably including schizophrenia and schizoaffective (SZA) disorder. Although clinical presentations are highly heterogeneous, the hallmark features predominantly involve disruptions in the content and structure of thought, perceptual disturbances, and disorganized speech, commonly referred to as positive symptoms. In addition, negative symptoms such as diminished emotional expression, avolition, alogia, anhedonia, and social withdrawal are also considered core aspects of these disorders [1,2].

The coexistence of affective features in patients presenting predominantly with psychotic symptoms has historically contributed to diagnostic ambiguity. Depressive symptoms include mood-related disturbances, such as sadness, low energy, and hopelessness, and they are particularly salient in SZA disorder. This complexity has, in turn, influenced the evolution of psychiatric classification systems, with the aim of establishing more precise and valid diagnostic boundaries. In recent revisions of these systems, efforts have been made to delineate schizophrenia more clearly from SZA disorder by introducing precise qualitative criteria, thereby addressing the limitations of broader and less specific definitions applied in earlier editions [3].

The term “schizoaffective disorder” was first introduced in the DSM-III in 1980, although it lacked clearly defined diagnostic criteria at that time [4]. The development of more specific criteria began in 1987 with the publication of the DSM-III-R [5]. Since then, successive revisions have aimed to reduce diagnostic uncertainty, particularly to differentiate patients with chronic psychosis who predominantly present with psychotic symptoms alongside affective features, but who do not fulfill the full diagnostic criteria for either schizophrenia or mood disorders. The DSM-5 further refined the diagnostic criteria for schizoaffective disorder by requiring that a major mood episode (depressive or manic) be present for most of the total duration of the illness. Additionally, the diagnosis still requires at least a two-week period of psychotic symptoms (e.g., delusions or hallucinations) occurring in the absence of any mood symptoms. In contrast, under DSM-IV criteria, the presence of a mood episode concurrent with psychotic symptoms, along with a minimum two-week period of psychosis without mood symptoms, was sufficient, even if mood symptoms were present only intermittently throughout the illness [6]. These clarifications have contributed to a more stable and operationalized diagnostic framework, wherein SZA disorder is no longer considered merely a diagnosis of exclusion, but rather a distinct clinical entity.

These refinements in diagnostic criteria have not only improved clinical clarity but also carry significant implications for prognosis and treatment outcomes. Psychotic spectrum disorders lead to significant personal, social, and functional impairments, making effective treatment strategies crucial for improving long-term outcomes [7]. While schizophrenia is traditionally characterized by a generally poor prognosis, SZA disorder, despite its chronicity, is often perceived in clinical practice as having a comparatively more favorable outcome. The presence of affective features in SZA disorder appears to be associated with better overall prognosis, distinguishing it from the typically more disabling trajectory observed in schizophrenia [8,9]. Although affective symptomatology is often regarded as a favorable prognostic factor in psychotic disorders, there remains a critical need for treatment approaches that adequately address both mood and psychotic symptoms in an integrated manner [10,11].

While pharmacological treatment remains the cornerstone of care for psychotic disorders, suboptimal therapeutic outcomes, adverse side effects, and the influence of psychological and social factors have increasingly highlighted the need for complementary psychosocial interventions. Among these, CBT has emerged as an evidence-based approach, demonstrating modest yet clinically meaningful effects in reducing symptom severity and improving overall functioning in individuals with psychosis [12].

CBT was initially developed by Aaron T. Beck as a structured, time-limited approach primarily targeting depression and anxiety disorders [13], with strong evidence of efficacy [14]. However, limitations associated with the traditional cognitive model, particularly its emphasis on cognitive restructuring, led to the evolution of CBT and the emergence of so-called third-wave cognitive therapies. These approaches incorporate novel techniques and offer a more holistic and experiential perspective, focusing on processes such as acceptance, commitment, mindfulness, and compassion. Third-wave therapies, including Acceptance and Commitment Therapy (ACT) [15,16], Metacognitive Therapy (MCT) [17], Mindfulness-Based Cognitive Therapy (MBCT) [18], and Compassion-Focused Therapy (CFT) [19], emphasize psychological flexibility and emotional regulation, moving beyond symptom reduction toward enhancing overall well-being [20].

Over the past few decades, considerable efforts have been made to adapt CBT for the treatment of more severe mental disorders, including schizophrenia and chronic mood disorders. These adaptations have primarily examined CBT as an adjunctive intervention, used in combination with pharmacotherapy [21,22], although there is limited evidence supporting its safety and effectiveness for individuals across the psychotic spectrum as a first-line treatment [23]. CBT for psychosis (CBTp) was initially developed to target persistent positive symptoms—such as delusions and hallucinations—by modifying dysfunctional beliefs and enhancing coping strategies [21,24,25,26,27]. Over time, its scope has expanded to include interventions addressing negative symptoms, social functioning, and emotional distress [28].

Both traditional CBT and third-wave therapies have been extensively studied in the context of psychotic disorders. A growing body of evidence supports their effectiveness in reducing symptom severity, improving functional outcomes, and promoting recovery and quality of life [14,29,30,31,32,33,34]. Importantly, traditional CBT models and third-wave approaches appear to target different dimensions of psychopathology, offering complementary mechanisms of therapeutic action [35,36,37,38,39].

SZA disorder, despite ongoing efforts to define it clearly, remains a diagnostically complex condition due to the coexistence of psychotic and mood-related symptomatology [10,40]. Although it shares substantial clinical overlap with schizophrenia, important differences have been identified in terms of age of onset, prognosis, treatment response, and cognitive functioning [41].

From a therapeutic perspective, the presence of affective symptoms in SZA disorder may shape the psychological processes targeted in CBT and influence treatment outcomes. Despite these differences, most CBT trials have primarily focused on individuals with schizophrenia or have grouped psychotic spectrum disorders together without distinction, thereby limiting the ability to draw conclusions about diagnosis-specific effects. Some findings suggest that comorbid affective symptoms—such as depression, anxiety, or mood instability—may moderate treatment response in CBTp, particularly by influencing engagement with cognitive restructuring techniques [42,43]. Furthermore, while differences in symptomatology—such as the prominence of mood symptoms in SZA disorder or the higher prevalence of perceptual disturbances in schizophrenia—may necessitate specific therapeutic adaptations [44,45], no systematic review to date has comprehensively examined the differential effectiveness of CBT across psychotic diagnoses characterized by varying affective symptom profiles. Consequently, despite the increasing clinical application of CBT across psychotic disorders, there remains a notable scarcity of studies specifically investigating its effectiveness in SZA disorder. Most clinical trials either exclude SZA disorder or aggregate it under broader psychotic spectrum categories, thereby limiting our understanding of diagnosis-specific treatment outcomes. Only a few RCTs have specifically targeted individuals with SZA disorder or reported outcomes stratified by affective symptom burden. This has led to an underrepresentation of mood-related factors in both efficacy evaluations and treatment development. In addition, most existing CBTp protocols are designed primarily for positive symptoms and may inadequately address depressive, negative, or mood-instability-related symptoms, which are often central to SZA disorder. Finally, the evolution of diagnostic criteria, particularly the shift from DSM-IV to DSM-5 definitions for SZA disorder, introduces inconsistencies in participant classification across studies, potentially affecting the validity of subgroup analyses. This gap is particularly significant given that SZA disorder presents a distinct clinical profile, characterized by the interplay of psychotic and affective symptoms, which may differentially influence responses to CBT.

The present systematic review aims to synthesize the existing evidence on the comparative effectiveness of CBT in individuals diagnosed with schizophrenia and SZA disorder, with particular attention to how affective symptoms influence treatment outcomes across distinct symptom domains (positive, negative, and depressive symptoms). Exploring treatment outcomes across these three symptom domains is critical. First, each domain represents distinct psychopathological processes and may respond differently to CBT. Second, depressive symptoms may interact with both positive and negative symptoms, influencing treatment engagement, cognitive flexibility, and overall functional recovery. Finally, evaluating outcomes across these domains allows for a more nuanced understanding of CBT’s therapeutic potential and limitations, especially in diagnostically complex conditions like SZA disorder, where all three symptom clusters may co-occur. By clarifying whether treatment outcomes vary by diagnosis and symptom profile, this study aims to (1) inform future tailoring of CBT interventions for psychotic disorders with affective features, (2) highlight the need for more diagnostically precise research designs, and (3) contribute to the development of more personalized, symptom-targeted treatment frameworks in clinical practice, especially within the psychosis spectrum.

## 2. Methods

The Preferred Reporting Items for Systematic Reviews and Meta-Analyses (PRISMA) guidelines were followed [46]. The review protocol was registered in the Open Science Framework (OSF) platform at https://doi.org/10.17605/OSF.IO/JERCU.

*Eligibility Criteria:* Studies that met the following criteria were included. The target population was adults that, at the time of the study, were diagnosed with affective or non-affective psychosis, as those are defined in commonly used diagnostic systems (DSM-IV, DSM-5, ICD-10, ICD-11) as schizophrenia spectrum disorders for the former and schizoaffective, bipolar, or unipolar mood disorders with psychotic features for the latter. Of particular interest in this latter group is the psychopathological trait of mood congruency in the psychotic experiences (delusions, hallucinations) of the people that received the interventions of interest.

The intervention of interest was any CBT modality that incorporated ACT principles, CFT, MT, or mindfulness principles, and eligible comparators were treatment as usual (TAU), waiting list, or other psychotherapies. Pharmacotherapy as a separate comparator group was excluded. Only randomized controlled trials (RCTs) were included. Case-control studies, cohort studies, and case reports or case series were thus not included in order to minimize bias from design sources as much as possible.

*Outcomes*: Primary outcomes included the post-treatment changes of positive and negative symptoms. A quantification of this change by any validated questionnaire (e.g., PANSS) was deemed eligible. Secondary outcomes included depressive symptoms, anxiety, and quality of life.

Only published articles were used, and unpublished data was not considered.

The MEDLINE and EmBASE databases, clinicaltrials.gov, and the CENTRAL Cochrane database were scanned for relevant documents from 2000 to 15 November 2024. The rationale for the date restriction is that most of these third-wave CBT methods were fully fledged after 2000.

The search strategy included the query of each database with the following algorithm:

(schizophrenia OR psychosis OR psychotic OR ‘psychotic depression’ OR ‘affective psychosis’ OR ‘depression with psychotic’ OR schizoaffective OR ‘depression with psychotic’ OR ‘bipolar disorder with psychotic’) AND (‘CBT’ OR ‘cognitive behavioral’ OR ‘acceptance and commitment’ OR ‘acceptance-commitment’ OR ‘acceptance commitment’ OR mindfulness OR ‘dialectical behavior’ OR ‘dialectical behaviour’ OR metacognitive) AND (randomized)

**Figure 1:**
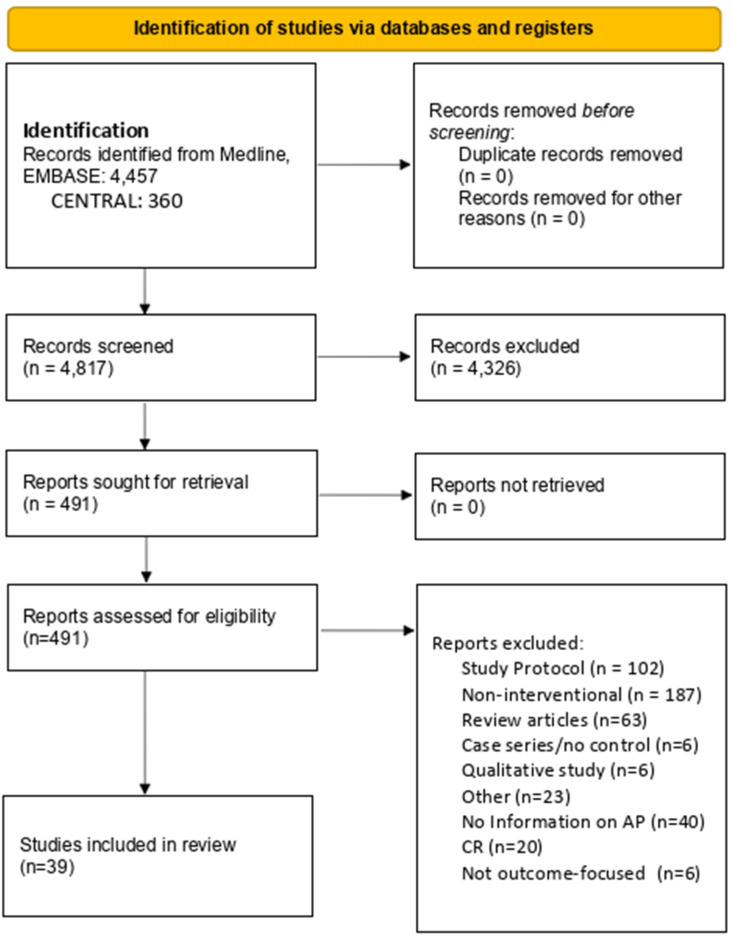
PRISMA 2020 flow diagram for included studies [46].

Titles and abstracts of the retrieved results were screened independently by two reviewers and assessed for eligibility. After identifying the most relevant articles, full-text articles were assessed in a second stage. For articles where there were disagreements, consensus was reached with the third author. The study selection process is documented using a PRISMA flow diagram (Figure 1).

*Data Extraction:* Data were extracted independently by three reviewers using a pre-designed extraction form. Extracted data included the following: Study characteristics: List of authors, year of publication, country, sample size, and study design. Participant details: Mean (standard deviation (SD)) age, sex (proportion of female participants), diagnosis of affective and non-affective psychosis participants, and mean duration of illness. Regarding the particular intervention as well as the comparator in each study, information on the modality, frequency and number of sessions, total duration, and delivery method of CBT was gathered (in-person or from a distance through technology, inpatient setting, or outpatient). For the outcomes, information was gathered on the instrument results at baseline and at each time point available, in whichever format was available (means, standard deviations, odds ratios, and risk ratios or hazard ratios for binary outcomes if those were opted for). Any discrepancies in data extraction were resolved through discussion among authors.

*Risk of Bias:* The risk of bias was assessed with the Cochrane Risk of Bias Tool (RoB 2.0) for RCTs. Information from each study was gathered on randomization, blinding, outcome measurement, and selective reporting, based on the published manuscripts and the descriptions the authors provided on their workflows.

*Data Synthesis:* We performed a narrative synthesis of the results. Where sufficient data were available, meta-analyses with the inverse variance-weighted method were conducted using R statistical software version 4.0.3. Heterogeneity was assessed using the I^2^ statistic and Cochran’s Q test, and if heterogeneity was present, a random-effects model was used to accommodate it.

The main analysis was conducted based on psychosis subtype, that is, on how CBT affects patients differently in the affective vs. the non-affective groups. The meta-regression model is represented by β = β0 + βAP × AP, where β0 is the intercept term of the pooled effect, βAP is the additional meta-regression term, and AP is the proportion of AP patients in each study. The βAP coefficient and the respective 95% CI were reported. The meta-regression models were compared with the random-effects models that do not incorporate the moderators with an AIC test. A total (any CBT modality, changed from protocol to incorporate any CBT modalities) and separate subgroup analyses (ACT, MCT, CFT, MBT) were planned.

*Publication Bias:* Publication bias was assessed in outcomes of at least 10 studies, and funnel plots and the formal statistic from the Egger’s test were used. After a reviewer’s suggestion, we performed a post hoc sensitivity analysis and removed small studies (*n* < 20) and repeated the meta-analysis and meta-regression analyses for positive, negative, and depressive symptoms.

## 3. Results

Through the literature search, a total of 4457 titles were identified, and the results of the search are presented in detail in Figure 1. A total of 39 studies met the inclusion criteria and were carried forward.

*Study Characteristics* Interventions that were represented were a range of CBT variants developed or used for study-specific goals, including CBT for psychosis, substance use in psychosis [47], relapse prevention in psychosis [48], adherence in psychosis [49], and technologically extended techniques [50] (Table 1).

A total of 422 patients with affective psychoses were included across the studies, with 241 (57.1%) of them in the treatment arm. Eight studies [23,51,52,53,54,55,56,57] reported a total number without specifying the AP patient distributions in the treatment and control arms.

*Patient Characteristics:* In Table 1, the patient characteristics are presented. Overall, young and male-skewed samples are observed, with mean ages ranging from 22.6 to 42.6 and a weighted mean age of 37.5 years, and in total 33% of patients were female. The mean proportion of post-secondary or higher education completion was 23.4% (Table 1). The proportion of AP was generally low to moderate, with a median of 17% of the included participants having AP. Six ACT studies [58,59,60,61,62] included AP cases. Most ACT studies had ≤30% AP participants in the groups, suggesting a primary focus on non-affective disorders. Likewise, for MCT, five studies contributed a total of 40 AP patients. CBT as a category contributed the most studies. There were two CBT studies in which AP patients comprised over 30% of the total number of patients [63,64]. Other trials included much lower rates of AP patients [65,66], showing the heterogeneity in study populations. Stratification by outcome of diagnosis was rare. Importantly, as can be seen in Table 1, control conditions were heterogeneous, with some comparisons using active treatments with a talking treatment component, such as befriending or psychoeducation, and others using treatment as usual.

**Table 1:** Study and patient characteristics for the included studies. Sample size: number of patients randomized to treatment and control conditions, with the number of patients with affective psychosis in parenthesis. MCT: metacognitive therapy; ISMI: internalized stigma of mental illness; DACOBS SC: Davos Assessment of the Cognitive Biases Scale—subjective cognitive subdomain; PANSS: Positive and Negative Syndrome Scale; ACT: acceptance and commitment therapy; SOFAS: Social and Occupational Functioning Assessment Scale; CBT: cognitive behavioral therapy; CR: cognitive remediation; WL: waiting list; TAU: treatment as usual; BD: bipolar disorder; MDD: major depressive disorder; AP: affective psychosis; CBSP: cognitive behavioral suicide prevention; MBT: mindfulness-based therapy; AIM-AT: acceptance-based, insight-inducing medication adherence therapy.

PMID	Author	Year	Country	Sample Size (AP)	Treatment and Control	Design Notes	Mean (SD) Age	Sex (% F)	Education
16648530	Baker, A. [53]	2006	Australia	49/55 (15)	CBT, TAU	Substance use	28.12	0.22	
30376124	Balzan, R.P. [67]	2019	Australia	27/27 (6/5)	MCT, TAU	PANSS General proxy for depression	37.21 (8.74)	0.41	11.41 years (2)
21106618	Barrowclough, C. [47]	2010	UK	129/118 (13/14)	CBT, TAU	24-month follow-up used	37.9 (9.7)	0.14	16.1 (1.8) left education
38908265	Chien, W.T. [62]	2024	Hong Kong	42/42 (8/7)	AIM-AT, TAU	Early-stage psychosis	27.1 (6.4)	0.31	0.24 university
33580033	Dellazizzo, L. [68]	2021	Canada	37/37 (9/8)	VRT, CBT	Both arms active treatment	42.5 (12.7)	0.24	12.2 (3.6)
18851771	Farhall, J. [69]	2009	Australia	45/49 (1/6)	CBT, TAU	Second follow-up used	32.9 (10.2)	0.41	0.12
37716893	Farrelly, S. [70]	2024	UK	11/10 (3/2)	CBT, TAU	Psychotic depression and BD in AP	40.5 (13.1)	0.29	0.24 university
24176646	Favrod, J. [71]	2014	Switzerland	26/26 (5/4)	MCT, TAU		36.7 (10.1)	0.35	0.12 post-secondary
34246324	Freeman, D. [72]	2021	UK	66/64 (13/11)	CBT, Befriending		41.6 (12.1)	0.40	
25468186	Freeman, D. [36]	2014	UK	15/15 (2/4)	CBT, TAU	12-week follow-up used	41.7 (12.3)	0.33	
26360083	Freeman, D. [42]	2015	UK	73/77 (5/6)	CBT, TAU	Six sessions	41.5 (11.5)	0.43	
33825827	Garety, P. [50]	2021	UK	161/171 (30/34)	CBT, TAU	24-week follow-up used	42.6 (11.6)	0.30	0.24 post-secondary
26352221	Gaudiano, B.A. [58]	2015	USA	6/7 (1/1)	ACT, TAU		50 (17.0)	0.54	14 (2.5)
15893293	Gaudiano, B.A. [51]	2006	USA	19/21 (5)	ACT, TAU		40 (10)	0.39	0.17 post-secondary
37716204	Gaudiano, B.A. [61]	2023	USA	23/23 (13/11)	ACT, TAU	Inpatients	40.03 (11.63)	0.48	11.5 (5.2)
22130905	Gleeson, J.F. [48]	2013	Australia	41/40 (6/2)	rpCBT, TAU	BPRS used for proxy	20.1 (3.1)	0.37	12.0 (2)
18005494	Jackson, H.J. [63]	2008	Australia	31/31 (10/10)	CBT, Befriending	BD and MDD in AP	22.3 (3.6)	0.27	0.13 in occupation
39610049	Katsushima, M. [49]	2025	Japan	12/12 (2/2)	CBT, TAU	Seven sessions	33.5 (10.8)	0.58	
23635846	Kråkvik, B. [66]	2013	Norway	23/22 (1/1)	CBT, TAU		36.4 (10)	0.36	
35485835	Lepage, M. [54]	2023	Canada	30/21 (11)	CBT, CR	SOFAS as proxy for depression	24.6 (4.4)	0.34	12 years (2.2)
22663901	Lincoln, T.M. [28]	2012	Germany	40/40 (6/7)	CBT, WL				
32994792	López-Navarro, E. [73]	2020	Spain	26/26 (6/5)	MBT, IRT		39.70 (9)	0.21	12 years (2)
30318868	MacDougall, A.G. [55]	2019	Canada	9/8 (1)	MAP, TAU		23.7 (NA)	0.24	0.06 post-secondary
30001930	Morrison, A.P. [74]	2018	UK	242/245 (28/20)	CBT, TAU		42.5 (10.6)	0.28	30/11/2000
27092862	Morrison, A.P. [52]	2016	UK	15/14 (1)	CT, TAU		34.3 (13.3)	0.21	
24508320	Morrison, A.P. [23]	2014	UK	37/37 (2)	CT, TAU		31.3 (12.5)	0.47	
28828697	Pos, K. [75]	2018	Netherlands	19/14 (2/1)	MCT, OT		23.3 (3.6)	0.20	higher secondary school most prevalent
27979820	Shawyer, F. [60]	2017	Australia	49/47 (14/9)	ACT, TAU	PANSS general proxy for depression	36.1 (9.1)	0.39	0.20 university
31129983	Sheaves, B. [64]	2019	UK	11/9 (5/4)	CBT, TAU		41 (12.5)	0.42	
31935529	Sönmez, N. [76]	2020	Norway	32/31 (7/3)	CBT, TAU	Six-month follow-up	27.9 (18–51)	0.41	0.35 in occupation
15259826	Startup, M. [77]	2004	Australia	47/43 (6/1)	CBT, TAU				
24853059	Tarrier, N. [78]	2014	UK	25/24 (8)	CBSP, TAU		34.9 (13.1)	0.37	0.17 post-secondary
22941746	van der Gaag, M. [65]	2012	Netherlands	98/103 (1)	CBT, TAU	Ultra-high-risk focus of patient cohort	22.8 (5.5)	0.51	13.9 years (2.7)
25066223	van Oosterhout, B. [79]	2014	Netherlands	75/79 (3/5)	MCT, TAU	24-month follow-up DACOBS SC proxy for depression	37.5 (9.9)	0.29	‘medium’ on average
21975193	White, R. [59]	2011	UK	14/13 (3/3)	ACT, TAU		34 (9.7)	0.22	0.15
29494866	Wood, L. [80]	2018	UK	15/15 (4/2)	CBT, Psychoeducation	ISMI proxy for negative	33.6 (12.9)	0.23	0.37
*Contributed to systematic review only*									
34470506	Wojtalik, J.A. [81]	2022	USA, Canada	58/44 (NA)	CET, enriched TAU		24.8 (5.5)	0.26	0.68
28166848	Ochoa, S. [82]	2017	Spain	65/57 (5/4)	MCT, TAU			0.30	0.23 post-secondary
26298541	López-Navarro, E. [83]	2015	Spain	22/22 (5/4)	MBI, CBT	26 weeks	38.8 (8.1)	0.17	0.25 left education after 18
29207980	Husain, M.O [84]	2017	Pakistan	18/18 (NA)	CBT, TAU				

### Primary Outcomes

Overall, 36 studies contributed quantitative data to one of the three analyses for positive, negative, or depressive symptoms, and four further studies contributed qualitative data. The key results are presented in Table 2.

Four studies contributed qualitative data. The study by Lopez-Navarro et al. [83] shows that there may be benefits in inhibitory control, but quantitative analysis was not performed, as post-treatment values for PANSS Positive, Negative, or General were not reported, nor were any sensible proxies estimable from the provided statistics. Two studies (by Hussain et al. [84] and by Ochoa et al. [82]) did not provide a tabulation of SZA disorder patients by treatment arm nor a total number. The results from Hussain et al. showed a lasting efficacy for positive symptoms only, and the results from Ochoa et al. [82] for MCT showed an improvement in both groups in a comparable manner, notably using an active control group (psychoeducation). Regarding MCT, there was some improvement in the cognitive domain that can be argued to relate to depressive symptomatology. The study by Wojtalik et al. [81] did not report the exact number of SZA disorder patients, and the results showed more reliable outcomes for cognition and no strong evidence for positive or negative symptoms.

**Figure 2:**
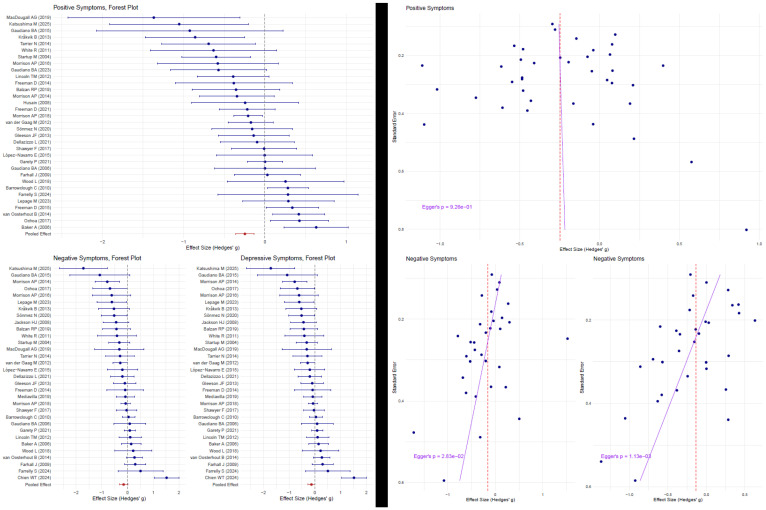
**Left** panel: forest plots of the standardized mean differences of CBT vs. control conditions for positive [23,28,36,42,47,48,49,50,51,53,54,55,58,59,60,61,65,66,67,68,69,70,73,74,76,77,78,79,80,82,84], negative [15,23,28,36,47,48,49,50,51,52,53,54,55,57,59,60,62,63,65,66,67,68,69,70,73,74,76,77,78,79,80,82], and depressive symptoms [23,28,47,48,49,50,52,53,54,55,57,58,59,60,62,63,65,66,67,68,69,70,72,73,74,76,77,78,79,80,82]. The blue points and lines represent individual effect estimates and corresponding 95% confidence intervals (CI) and the red lines represent pooled estimates and corresponding 95% CIs. **Right** panel: Funnel plots for positive, negative, and depressive symptoms. The points represent the inverse standard error of the effect size of each study, the blue lines represent the fitted lines from Egger’s regression, and the red dashed lines represent the pooled effect sizes for each outcome.

**Figure 3:**
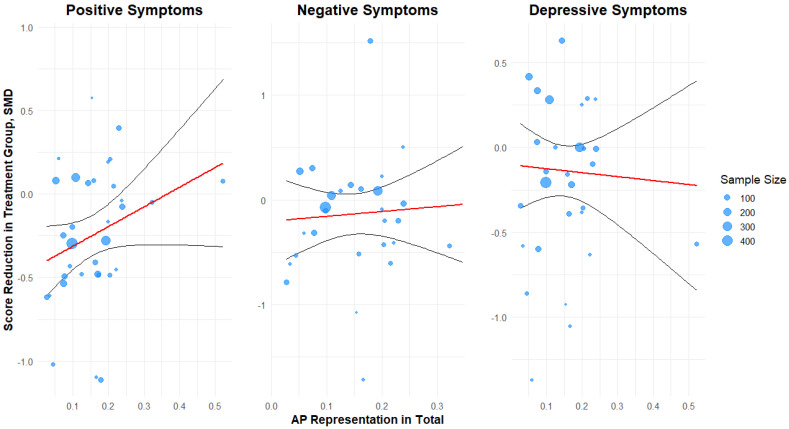
Scatterplot for the potential role of total representation of affective psychosis (AP) as a moderator of the effects of CBT on different symptom domains (positive symptoms, negative symptoms, and depressive symptoms). Each point represents the point estimate of each study, each red line represents the mean predicted score reduction based on the fitted model with AP representation as a predictor, and the black lines represent the corresponding 95% confidence intervals. Different sample sizes in each individual RCT are represented by different point sizes. SMD: standardized mean difference. Negative values favor CBT.

The random-effects model without a moderator suggested a statistically significant moderate overall effect of the intervention (SMD = −0.25, 95% CI: −0.36 to −0.14) with substantial heterogeneity between studies (I^2^ = 54.5%) (Figure 2). The meta-regression analysis indicates a positive association of the proportion of AP individuals in both arms with positive symptom reduction (β = +0.10 SMD per 10% increase in AP, 35 studies), suggesting that studies with more SZA disorder patients in total show a smaller benefit of CBT. This finding did not reach statistical significance (95% CI: −0.03, 0.22, *p* = 0.13) (Figure 3). Inclusion of the additional moderator term in the mixed-effects model explained an additional 1% of the variance and still indicated substantial residual heterogeneity (I^2^ = 55.03%). Effect sizes suggestive of a protective effect of CBT on negative symptoms (−0.15, 95% CI −0.32, 0.01, *p* = 0.07, 32 studies) and depression (β = −0.13, 95% CI: −0.27, 0.01, *p* = 0.06, 33 studies) were estimated, albeit without reaching statistical significance at the pre-specified level (Figure 2). The meta-regression analyses revealed a numerically smaller association of the total proportion of AP individuals and CBT magnitude of effect on negative symptoms (β = +0.052 SMD per 10% increase in AP (−0.18, +0.28), R^2^ = 0.0%, *p* = 0.73). In both the RE model and the mixed effects model, substantial residual heterogeneity was present (I^2^ =76.86% and 79.9%, respectively). Similarly, a flat association was estimated in depressive symptoms (β = −0.02 (−0.18, +0.14) SMD per 10% increase in AP, R^2^ = 0.02%, *p* = 0.78), with high residual heterogeneity (I^2^ = 68.2%) (Figure 3).

No evidence for funnel plot asymmetry was detected for the positive symptoms, as assessed via Egger’s test (z = −0.39, *p* = 0.69) (Figure 2). On the contrary, there was evidence for asymmetry in the negative and depressive symptom outcomes (*z* = −2.15, *p* = 0.03 and *z* = −3.02, *p* = 0.003, respectively), which could be interpreted as potential publication bias (Figure 2). In the post hoc sensitivity analysis that removed small studies (*n* < 30), a total of six studies (those by Gaudiano et al. [58], Katsushima et al. [49], Farrelly et al. [70], White et al. [59], Sheaves et al. [64], MacDougall AG [55], and Morrison et al. [52]) were excluded. The results show that βAP retains its positive but still not statistically significant association (βAP, positive (SE) 1.01 (0.65), *p* = 0.119). The coefficient changes most for depressive symptoms; a non-significant improvement of CBT in studies with higher AP representation is estimated (βAP, depressive (SE): −0.48 (0.75), *p* = 0.53). The meta-regression estimate for negative symptoms increases in the selected subsample of larger studies, favoring lower AP representation, and remains non-significant (βAP, negative 0.29 (1.19), *p* = 0.805).

**Figure 4:**
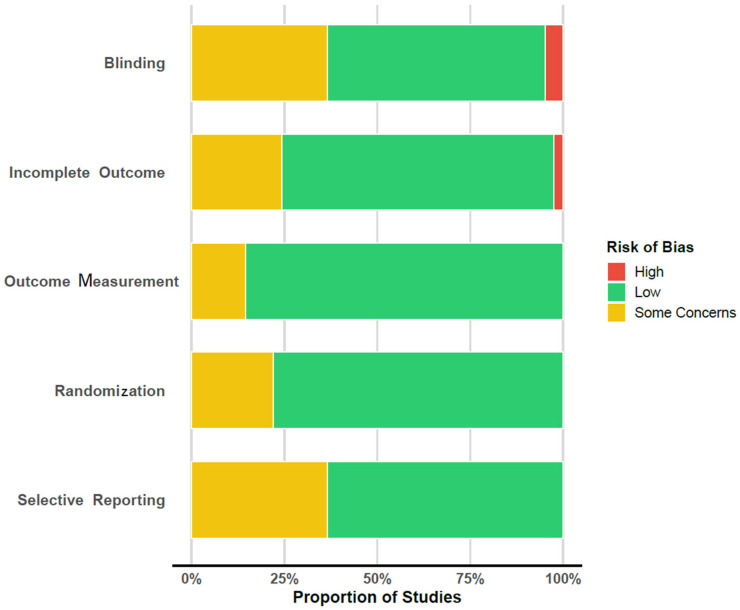
Risk of bias assessment. Each colored line segment represents the proportion of studies that received the respective risk of bias judgement.

*Risk of Bias Assessment:* Individual-study and summary risk of bias assessments are presented in Figure 4 and Table 3 respectively. As anticipated, blinding was one domain where there were inherent difficulties in delivering a treatment without providers and patients. Even if double-blind studies are not feasible, there were still some studies where unblinding or the risk of unblinding raised some concerns. Also, there were varying degrees of blinding assessors. Regarding incomplete outcomes, there were two studies with relatively high dropout rates that could possibly lead to attrition bias, such as [81], where almost half of the originally enrolled participants dropped out. Regarding outcome measurement, there were some concerns with one study for the handling of secondary outcomes that was conditional on the transition to psychosis status, thereby excluding those that did not transition to psychosis status but whose outcomes still changed [65]. For another study where interrater reliability for outcome assessments was unclear for some outcomes [79], there was a possibility of potential subjective outcome changes due to unblinding and expectancy effects for those in the treatment arm, although these measures were not chosen in the reported analyses [59]. Regarding selective reporting, there was good alignment of methods and results reporting within the published papers. There were some concerns for those studies where a pre-registered protocol was not explicitly shared in the methods section [23,59,60,64,65,75,76,77,78,79,80,81,82,85].

*Subgroup Analysis:* A total of four studies assessed MCT interventions, five studies assessed ACT or ACT-inspired interventions, and two studies reported results on mindfulness. Due to this paucity of studies (<10 as per the rule of thumb in meta-regression analyses) for each specific modality, meta-regression models were not fitted and the results are explored qualitatively. For MCT, SZA disorder proportions ranged from 9.1% to 20.4%, for ACT from 12.5% to 52.2% and for mindfulness from 5.9% to 20.5%. Both mindfulness studies estimated modest improvements in negative and depressive symptoms but not in positive symptoms. ACT was favored for positive and depressive symptoms but not with large effect sizes; of note, one study where half of the participants had AP showed equivalence in positive outcomes for the treatment and control groups. MCT showed a somewhat better positive outcome profile in three of four studies [67,71,75,79], and similar outcomes in both arms for depressive and negative symptoms.

## 4. Discussion

This study examined the comparative effectiveness of various CBT modalities across schizophrenia spectrum disorders, with a particular focus on the inclusion and proportion of individuals with SZA disorder. Overall, CBT demonstrated a statistically significant moderate effect in reducing positive symptoms, with substantial heterogeneity across studies, as shown by previous studies. Notably, our meta-regression results revealed a non-significant trend indicating that a higher proportion of individuals with SZA disorder in study populations was associated with a smaller benefit of CBT for positive symptoms. No significant associations were identified for negative or depressive symptom outcomes.

Previous meta-analyses reviewing CBT for schizophrenia spectrum disorders have also included SZA disorder but did not specifically target it as a potential moderator. There has been extensive reporting on affective symptoms, which are particularly important for SZA disorder, but to our knowledge there has been no explicit stratification by diagnostic status [86,87].

The present finding of decreased CBT effectiveness with increasing representation of patients with SZA disorder is broadly consistent with prior observations suggesting that individuals with affective symptomatology may respond differently to CBT than those with psychotic disorders without predominant affective features [88,89].

One possible explanation lies in the CBTp protocol itself. CBTp primarily targets delusional beliefs, hallucinations, and related cognitive distortions, but may insufficiently address the affective components such as mood instability, dysphoria, and comorbid anxiety, which are central to SZA disorder [24,27,86]. Consequently, treatment approaches that do not explicitly integrate affect-focused techniques may yield suboptimal outcomes in affective symptomatology.

In addition, patients with SZA disorder often present with higher functional variability, more frequent episodes, and fluctuating insight, which may impair therapeutic engagement or limit the long-term outcomes of CBT gains [10,11]. The presence of mood episodes may also affect cognitive flexibility, goal-setting capacity, and session-to-session continuity, further reducing the efficacy of CBT protocols.

Another possible interpretation of the findings arises from the heterogeneity in the CBT interventions themselves. The included studies employed diverse CBT variants with different treatment targets, including substance use in psychosis, relapse prevention, medication adherence, and even technology-assisted modalities. These adaptations, although clinically relevant, likely differ in their mechanisms of action and are not equally applicable across diagnostic subtypes.

Interestingly, our analysis revealed no significant associations between the proportion of individuals with affective symptoms and the effectiveness of CBT for negative or depressive symptoms. This finding requires careful interpretation, especially considering that affective symptoms and negative symptoms often overlap or are difficult to distinguish. From a clinical perspective, the lack of a significant association between the affective subgroups proportion and the treatment effects on negative and depressive symptoms may indicate that current CBT approaches do not adequately address the affective and motivational deficits commonly seen in these patients.

In the case of negative symptoms, these are often secondary to chronic affective disturbance, social isolation, or cognitive disorganization—factors that are not consistently or directly addressed by standard CBTp [90]. Moreover, the therapeutic strategies commonly employed—such as cognitive restructuring, reality testing, and challenging delusional beliefs—may have limited impacts on the more persistent dimensions of negative symptomatology, such as avolition and anhedonia [91]. Finally, it should be noted that the instruments used to measure negative symptoms may lack the sensitivity to capture subtle therapeutic gains, particularly when these symptoms are often presented as secondary outcomes [92].

As for depressive symptoms, we might have expected a stronger CBT effect among individuals with affective features, given CBT’s cognitive focus on mood-related distortions and dysfunctional beliefs [13,14]. However, our analysis did not support this hypothesis. This discrepancy may reflect a mismatch between the therapeutic content of the CBT protocols used and the specific affective needs of participants. Consequently, the impact of these interventions on depressive symptomatology may have been limited, even for patients in whom such symptoms were prominent. Alternatively, it is possible that depressive symptoms in SZA disorder are driven by mechanisms that are less responsive to cognitive interventions—such as underlying neurobiological vulnerability, mood instability, or persistent psychosocial stressors—thereby requiring more integrative or pharmacologically oriented approaches [10,11].

Taken together, the interaction between diagnostic subtype and CBT focus may act as a confounding factor in assessing comparative effectiveness. The trend toward reduced efficacy in SZA disorder may not reflect an inherent resistance to CBT but rather a mismatch between intervention content and patient needs.

Our findings carry several important theoretical and practical implications for future research and clinical practice. From a theoretical point of view, we assume that CPTp does not result in effective outcomes, regardless of diagnostic boundaries. While CBTp is well-established for treating psychotic symptoms, the lack of differential efficacy in SZA disorder suggests that affective comorbidity may not enhance responsiveness to cognitive interventions. This nuance prompts a reconsideration of how symptom dimensions interact with cognitive targets in psychotherapy. The results support a more dimensional, rather than categorical, approach to conceptualizing psychosis, emphasizing symptom profiles over rigid diagnostic labels. This reinforces the need for transdiagnostic frameworks that recognize heterogeneity in symptom presentation and treatment response. From a clinical point of view, there is a clear need for the development and rigorous evaluation of integrated CBT protocols that address both psychotic and affective symptom domains. Such approaches might incorporate techniques from traditional CBT for depression (e.g., behavioral activation) as well as third-wave interventions, which are designed to address mood dysregulation, self-stigma, and interpersonal dysfunction [29,30,32,33,51,58,61,83]. Clinicians should consider adopting individualized models that account for the presence of prominent affective instability, motivational deficits, and depressive cognitions, which are often under-targeted in standard CBTp, in addition to psychotic features. In this context, CBT may be most effectively deployed as a targeted adjunctive intervention, complementing other therapeutic modalities such as social skills training, peer support programs, or pharmacological augmentation. Such multidimensional treatment frameworks may be especially valuable in SZA disorder, where symptom complexity and variability often underline the need for flexible and personalized therapeutic strategies. Furthermore, the diagnostic ambiguity surrounding SZA disorder underscores the importance of precise assessment and reporting in both clinical practice and research. Future trials should stratify participants based on affective symptom severity and duration and align diagnostic criteria with current nosological standards (DSM-5). This will facilitate more accurate conclusions regarding treatment efficacy and inform personalized intervention planning. Ultimately, enhancing specificity in both diagnosis and intervention may lead to improved outcomes for subgroups traditionally considered less responsive to standard psychosis treatments.

Consequently, future research should prioritize several key areas. First, diagnosis-specific trials that treat SZA disorder as a distinct clinical entity are needed, employing rigorous diagnostic procedures aligned with DSM-5 criteria. Second, studies using symptom-dimension-based designs should explore how mood symptoms, negative symptoms, and cognitive dysfunction mediate or moderate CBT outcomes. Third, tailored CBT interventions that incorporate mood-focused modules and are responsive to the specific clinical needs of individuals with affective psychoses are recommended. Finally, individual participant data (IPD) meta-analyses should be considered, as they allow for modeling of treatment effects at the individual level and minimize ecological bias inherent in study-level data. Such efforts would enhance diagnostic precision, increase treatment specificity, and improve the overall clinical utility of CBT across the broader spectrum of psychotic disorders. The present analyses reveal moderate to high heterogeneity across outcomes, which may reflect differences among patient subgroups, intervention characteristics, and study designs.

Regardless of specific diagnostic classification, we argue that the inclusion of individuals with affective psychosis serves as a valid proxy for our primary variable of interest: the prominence of mood symptoms. While our meta-regression models incorporated the proportion of individuals with SZA disorder as a moderator, a large proportion of variance remained unexplained. This suggests that other factors, such as baseline symptom severity, treatment adherence, therapist expertise, or psychiatric comorbidities, may meaningfully influence outcomes.

The risk of bias assessment highlighted key challenges inherent in psychotherapeutic trials, especially regarding blinding and attrition. Although outcome assessors were blinded in several studies, performance and detection bias cannot be ruled out. The presence of funnel plot asymmetry in negative and depressive symptom outcomes raises the possibility of publication bias, particularly the selective reporting of favorable CBT outcomes.

### Limitations

Our findings should be interpreted considering several limitations. First, the classification of psychosis with affective features was based on variable reporting practices across studies. In many cases, trials did not clearly report the distribution of participants with affective psychosis across treatment arms, limiting the precision of subgroup analyses. Second, the inclusion of diverse CBT variants, ranging from standard CBTp to condition-specific adaptations, introduced clinical heterogeneity, which may have obscured specific treatment effects. Third, although we attempted to statistically adjust for the proportion of participants with SZA disorder in each study using meta-regression, residual confounding from unmeasured variables (e.g., treatment fidelity, severity of baseline symptoms, or comorbidities) remains possible.

A further limitation relates to the evolution of diagnostic criteria for SZA disorder. Given that our literature review spans studies published since 2000, a substantial portion of the included trials were conducted prior to the DSM-5 revision (2013), implying that some individuals diagnosed with SZA disorder under DSM-IV criteria may not fulfill the more stringent diagnostic criteria currently in place. This temporal inconsistency may have contributed to diagnostic heterogeneity and misclassification. However, we argue that this limitation does not affect the conceptual basis of our primary research question regarding the potential moderating role of mood symptomatology. Our focus was not only on formal diagnostic labels but also on the proportion of participants with prominent affective features.

An additional methodological limitation concerns our reliance on aggregate (study-level) data. Ideally, the research question posed here would be addressed through an individual participant data (IPD) meta-analysis, which would allow for direct modeling of diagnostic status and symptom profiles. Meta-regression at the study level is inherently vulnerable to ecological fallacy [93], whereby group-level associations may not accurately represent individual-level interactions. Therefore, while we did not observe statistically significant moderation effects by diagnostic category, it remains premature to rule out meaningful diagnostic influences at the individual level.

## 5. Conclusions

In conclusion, our findings suggest that the effectiveness of CBT in individuals with SZA disorder remains unclear and may be lower than in those with psychotic disorders without prominent mood symptoms. While CBT is generally effective for psychosis, its impact appears less consistent when affective symptoms are present.

Future research should prioritize clearer diagnostic definitions and consider integrating mood-focused strategies into CBT protocols for psychosis. Studies should also report diagnostic subtypes and symptom profiles more consistently and include subgroup analyses based on mood symptom severity. This approach could improve our understanding of how CBT works across different psychosis presentations and help tailor interventions more effectively. In addition, mechanism-focused studies that explore CBT outcomes in SZA disorder are critical to specify intervention targets.

## Figures and Tables

**Table 2:** Summary of key results. Overall effect size: standardized mean difference, with more negative values suggesting larger decreases in symptoms in the CBT groups; association with AP: percent change in SMD per 10% increase in affective psychosis patients in the sample (higher values show lower efficacy); publication bias: Egger’s regression results (z coefficient and *p*-value).

Symptom Domain	Overall Effect Size	Association with AP (% per 10%)	Explained Variance (R^2^)	Residual Heterogeneity (I^2^)	Publication Bias
Positive Symptoms	−0.25 (−0.36 to −0.14, *p* < 0.001)	+0.10 (−0.03 to +0.22, *p* = 0.13)	1%	55.03%	z = −0.39, *p* = 0.69
Negative Symptoms	−0.15 (−0.32 to +0.01, *p* = 0.07)	+0.05 (−0.18 to +0.28, *p* = 0.73)	0%	76.86%	z = −2.15, *p* = 0.03
Depressive Symptoms	−0.13 (−0.27 to +0.01, *p* = 0.06)	−0.02 (−0.18 to +0.14, *p* = 0.78)	0%	68.2%	z = −3.02, *p* = 0.003

**Table 3:** Risk of bias in individual studies. Green: low risk of bias, Yellow: moderate risk of bias, Red: high or unclear risk of bias.

Author	Year	Randomization	Blinding	Incomplete Outcome	Outcome Measure	Selective Reporting
Baker, A. [53]	2006	low	low	low	low	low
Balzan, R.P. [67]	2019	low	moderate	moderate	low	NP/PNI
Barrowclough, C. [47]	2010	low	low	moderate	low	NP/PNI
Chien, W.T. [62]	2024	low	moderate	low	low	low
Dellazizzo, L. [68]	2021	low	low	low	low	low
Farhall, J. [69]	2009	moderate	high	moderate	moderate	low
Farrelly, S. [70]	2024	low	low	low	low	low
Favrod, J. [71]	2014	low	low	low	low	low
Freeman, D. [72]	2021	low	low	low	low	low
Freeman, D. [36]	2014	low	low	low	low	low
Freeman, D. [42]	2015	low	low	low	low	low
Garety, P. [50]	2021	low	low	moderate	low	NP/PNI
Gaudiano, B.A. [58]	2015	low	low	low	low	NP/PNI
Gaudiano, B.A. [51]	2006	moderate	moderate	low	moderate	low
Gaudiano, B.A. [61]	2023	low	moderate	low	low	low
Gleeson, J.F. [48]	2013	moderate	low	low	low	low
Husain, M.O. [84]	2017	low	low	low	low	low
Jackson, H.J. [63]	2008	low	low	low	low	NP/PNI
Katsushima, M. [49]	2025	low	low	low	low	low
Krakvik, B. [66]	2013	moderate	high	moderate	moderate	low
Lepage, M. [54]	2023	low	moderate	low	low	NP/PNI
Lincoln, T.M. [28]	2012	low	moderate	low	low	NP/PNI
López-Navarro, E. [83]	2015	low	low	low	low	NP/PNI
López-Navarro, E. [73]	2020	low	low	low	low	low
MacDougall, A.G. [55]	2019	moderate	moderate	low	low	low
Morrison, A.P. [74]	2018	low	low	low	low	low
Morrison, A.P. [52]	2016	low	low	low	low	low
Morrison, A.P. [23]	2014	low	low	low	low	low
Ochoa, S. [82]	2017	moderate	moderate	low	low	low
Pos, K. [75]	2018	low	moderate	moderate	low	NP/PNI
Shawyer, F. [60]	2017	low	low	low	low	low
Sheaves, B. [64]	2019	low	low	low	low	low
Sonmez, N. [76]	2020	low	low	low	low	NP/PNI
Startup, M. [77]	2004	moderate	moderate	moderate	low	NP/PNI
Tarrier, N. [78]	2014	low	low	low	low	low
van der Gaag, M. [65]	2012	low	low	low	low	moderate (QoL Scale)
van Oosterhout, B. [79]	2014	low	low	moderate	low	NP/PNI
White, R. [59]	2011	low	low	low	low	NP/PNI
Wojtalik, J.A. [81]	2022	moderate	low	moderate	low	low
Wood, L. [80]	2018	low	low	high	moderate	low
